# Veterinary Treatment Approach and Antibiotic Usage for Clinical Mastitis in Danish Dairy Herds

**DOI:** 10.3390/antibiotics10020189

**Published:** 2021-02-15

**Authors:** Jensine Wilm, Line Svennesen, Esben Østergaard Eriksen, Tariq Halasa, Volker Krömker

**Affiliations:** 1 Section for Animal Production, Nutrition and Health, Department of Veterinary and Animal Sciences, University of Copenhagen, Grønnegårdsvej 2, 1870 Frederiksberg, Denmark; jwilm@sund.ku.dk (J.W.); esbene@sund.ku.dk (E.Ø.E.); 2 Section for Animal Welfare and Disease Control, Department of Veterinary and Animal Sciences, University of Copenhagen, Grønnegårdsvej 8, 1870 Frederiksberg, Denmark; line.svennesen@sund.ku.dk (L.S.); tariq.halasa@sund.ku.dk (T.H.)

**Keywords:** dairy cattle, mastitis treatment, antibiotic stewardship, veterinarian, survey, Denmark

## Abstract

Danish veterinarians’ treatment approach and use of antibiotics for clinical mastitis were investigated through a web-based questionnaire. The objective of the study was to describe and evaluate how the clinical mastitis treatment practice in Danish dairy herds corresponds to evidence from the literature and legislative requirements, in order to suggest directions for improvements and approaches encouraging the prudent use of antibiotics. In total, 174 veterinarians working with cattle received the questionnaire and 85 (48.9%) completed it. Their answers suggested that the Danish treatment approach for clinical mastitis generally relies on combined systemic and intramammary antibiotic administration (92% would use this often or always) and almost always includes supportive treatment with nonsteroidal anti-inflammatory drugs (99% would use it often or always in combination with antibiotic therapy). While collecting milk samples in order to target treatment towards pathogens is a priority in the legislation and for veterinarians, the direct application seems hindered due to the waiting time with the currently used analysis practice. Consequently, 91% reported that they would start treatment immediately after clinical examination often or always. The results of this investigation show that there is a potential for improvement in targeting treatments towards the causative pathogen by encouraging methods that allow for a more rapid reliable pathogen determination. When this issue has been addressed, the available evidence on the best treatment practice of Gram-negative-caused mastitis cases can be applied properly, reducing the volume of antibiotic treatments with limited expected effect. Additionally, investigating the potential of reducing combined administration to only intramammary treatment in Gram-positive cases could be a further step towards a more prudent antibiotic strategy.

## 1. Introduction

A growing societal concern has put emphasis on the prudent use of antibiotics to prevent the development of antibiotic resistance worldwide, thus urging both human and veterinary medicine to look for new solutions [1]. This also involves the Danish dairy industry, where the most common cause of antibiotic use in adult cows is mastitis [2]. Many recommendations have been made to promote the prudent use of antibiotics in relation to mastitis treatment, without compromising animal welfare [3]. Some of the best known recommendations include the following: the choice of treatment should be supported by knowledge about the etiology from milk sample analysis; targeted application of narrow-spectrum antibiotics is preferable; and the supportive use of nonsteroidal anti-inflammatory drugs (NSAIDs) is recommended [4,5,6]. Danish cattle production is modern and efficient and mainly specializes in dairy production, with 568,400 dairy cows in 2020 [7]. The average dairy herd size has been reported to be 227 cows, with an average performance of 11,042 kg energy-corrected milk per cow and an average bulk tank somatic cell count of 202,000 cells/mL [8]. Danish authorities have used the recommendations from the literature to build legislation that encourages the use of simple penicillin in mastitis treatment and requires the analysis of milk samples to justify the use of broad-spectrum antibiotics [9]. Furthermore, the use of fluoroquinolones has been almost completely phased out [9], while third- and fourth-generation cephalosporines have been voluntarily banned by the cattle industry [10]. In addition, organic farmers recently decided that all cases of clinical mastitis in organic herds should only be treated with simple penicillin and supportive treatment during the lactation period [11]. This leaves Danish cattle veterinarians with limited options at their disposal, but they must still make a series of decisions from diagnosis through to the initiation of treatment. These decisions could be about steps taken to detect pathogens and formulation of a treatment protocol, including selection of therapeutic substance(s) and duration and route of administration. Since 2010, all Danish dairy farms with more than 100 cows have had mandatory veterinary advisory service contracts (VASC) that require them to have regular veterinary consultancy visits [12]. The vast majority of Danish dairy herds (over 90%) have >100 cows, which means that they will have a VASC [7].

The prescription and administration of antibiotics to cattle is by default reserved for veterinarians in Denmark. However, farms with a VASC can obtain an optional module 1 or 2 extension, where staff can attend courses that will give them permission to carry out mastitis treatment on their own animals. The permission can either be limited to only cover follow-up treatment, after a veterinarian has initiated a treatment (module 1), or it can cover the entire treatment, following a pre-formulated treatment protocol for a specified diagnosis provided by the veterinarian (module 2). On many farms, the veterinarian will thus have an advisory role and will be responsible for formulating a list of clear clinical criteria and a treatment protocol for the farmer to follow. The farmer can collect the medicine prescribed by the veterinarian for his herd directly from the pharmacy or from the veterinarian. Organic farms do not participate in the module agreements although they are covered by the VASC system, so in principle veterinarians will carry out all treatments at these farms. Cattle veterinarians will also be called out to treat cases of mastitis at the remaining smaller farms or those without module 1 or 2 extensions, as well as in very severe cases on all farms. Farmers are, by principle, not allowed to administer any mastitis treatment intravenously, regardless of the VASC module status. There are generally two different main diagnoses for clinical mastitis used in Denmark: “common mastitis” and “acute mastitis”. While “common mastitis” generally refers to mastitis caused by Gram-positive agents, a diagnosis of “acute mastitis” is used for suspected Gram-negative agents. Some veterinarians may also distinguish between the two based on the severity of clinical symptoms, where a diagnosis of “common mastitis” aims to cover mild and moderate cases, while “acute mastitis” refers to severe cases.

The objective of the study was to describe and evaluate how the clinical mastitis treatment practice in Danish dairy herds corresponds to evidence from the literature and legislative requirements, in order to suggest directions for improvements and approaches encouraging the prudent use of antibiotics. This was based on the hypothesis that the context must be understood and potential incongruences need to be identified, in the aim of proposing meaningful adjustments.

## 2. Results

### 2.1. Sample Population

The questionnaire was distributed to *n* = 174 veterinarians who prescribed antibiotics for cattle. Of these, *n* = 85 (49%) participated and responded to the questionnaire: 46 women (54%) and 39 men (46%). The male respondents had generally worked with cattle for a longer period, which was closely correlated with age. Most of the respondents (81%) were located in Jutland, which is also the region of Denmark with the densest cattle population. Approximately half of the respondents (47%) reported that they treated >8 cases of mastitis per month, 28% treated between 4 and 8 cases and 25% treated <4 cases per month.

### 2.2. Considerations in the Decision-Making Process

**Figure 1:**
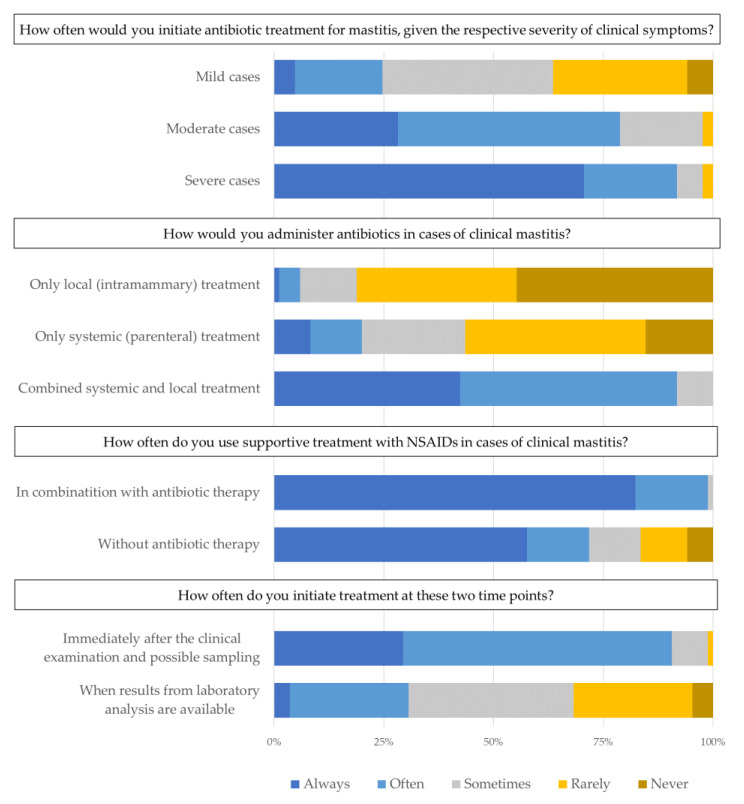
Answers from 85 Danish veterinarians to closed questions about treatment decisions in cases of clinical mastitis.

Respondents were asked to list what aspects they generally included in their decision-making process at the time of initiating a treatment for clinical mastitis, as shown in Table 1. The most popular answers on the list were clinical signs in the milk and udders and the general condition of the cow (95%; *n* = 81 of the respondents included these in their list of answers). These were followed by knowledge of the bacteriological state of the herd, which covers experiences with pathogens causing problems previously within the same herd, and factors related to the cow (65%; *n* = 56, and 61; *n* = 52, respectively). Open answers that 6% of respondents (*n* = 5) added to the list were time, whether the farmer was on site and preventing subclinical cases. When we asked the respondents how often they would initiate antibiotic treatment at different stages of mastitis severity using a Likert scale (Figure 1), the majority answered that they would often or always initiate treatment in severe or moderate cases (92%; *n* = 78, and 79%; *n* = 67, respectively). This was in contrast to mild cases, where only 25% (*n* = 21) would often or always initiate antibiotic treatment.

### 2.3. Route of Administration

When asked about the chosen route of administration for antibiotics prescribed for clinical mastitis in general, the majority of respondents (92%; *n* = 78) answered that they often or always used “a combination of local and systemic treatment”, while only 20% (*n* = 17) said that they often or always used “systemic treatment alone” and only 6% (*n* = 5) said that they often or always used “local treatment alone” (Figure 1).

### 2.4. Use of NSAIDs

Almost every respondent (99%; *n* = 84) said that they would often or always use “NSAIDs in combination with antibiotic treatment”, while 72% (*n* = 51) said that they would often or always use “supportive treatment with NSAIDs alone” (Figure 1) in cases of mastitis where they did not use antibiotics. The reported use of other supportive treatments was generally much lower (data can be found in the Appendix A, Table A4).

### 2.5. Milk Sampling and Treatment Initiation

When asked at what point in time they would initiate antibiotic treatment (Figure 1—before or after diagnostic results are available), the majority (91% or *n* = 77) answered that they would often or always start the treatment immediately. None of the respondents said that they would never start immediately. However, this seems to conflict slightly with the 4% (*n* = 3) of respondents who answered they always wait to start the treatment “when sample results are available”. When asked which factors are involved in the decision to take milk samples (Table 2), “legislation” was the most popular option in the list of answers (73% or *n* = 62), followed by 52% (*n* = 44) listing the “severity of clinical signs”. Interestingly, 39% of respondents (*n* = 33) chose to add another option, with *n* = 30 indicating that they always take milk samples as a general rule. The remaining three added suspected *Mycoplasma* spp. and cell count problems as reasons.

### 2.6. Pathogen-Specific Targeted Treatment

The respondents were asked whether they target mastitis treatment toward a specific pathogen before the sample results are available. Only 2% replied that they never target their treatment strategy. When respondents were asked about what factors they base their tentative diagnosis on before sample results are available (Table 3), 93% (*n* = 79) listed the option “severity of the clinical signs” and 86% (*n* = 73) listed “the appearance and/or smell of mastitic milk”. “Knowledge of previous cases of mastitis at the same farm” was listed by 68% (*n* = 58) of respondents, while 58% (*n* = 49) listed “the rate at which clinical symptoms appear” and “experience of previous treatment success at the same farm”.

### 2.7. Veterinarian Protocols for Treatments Conducted by Farmers

**Figure 2:**
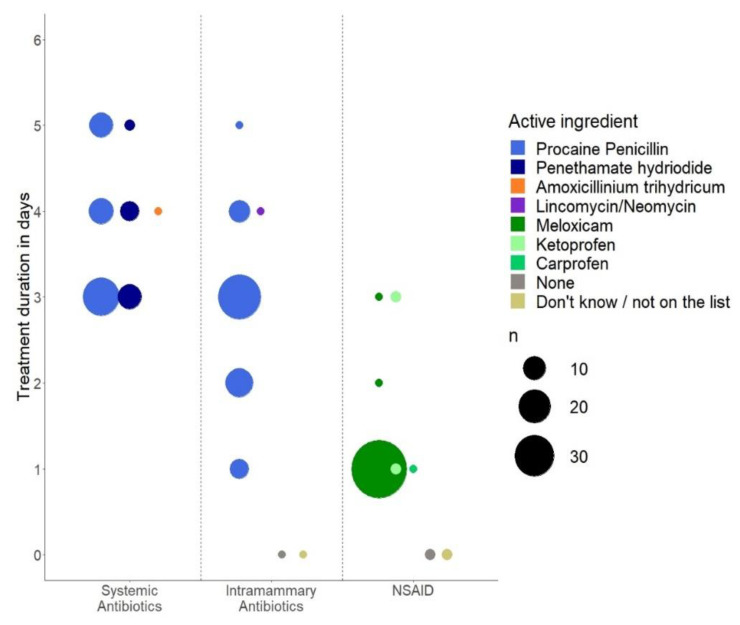
Prescription protocol of systemic antibiotics, local antibiotics and nonsteroidal anti-inflammatory drugs (NSAIDs) used often or always by Danish veterinarians (*n* = 70) for a herd diagnosis of “common mastitis”.

**Figure 3:**
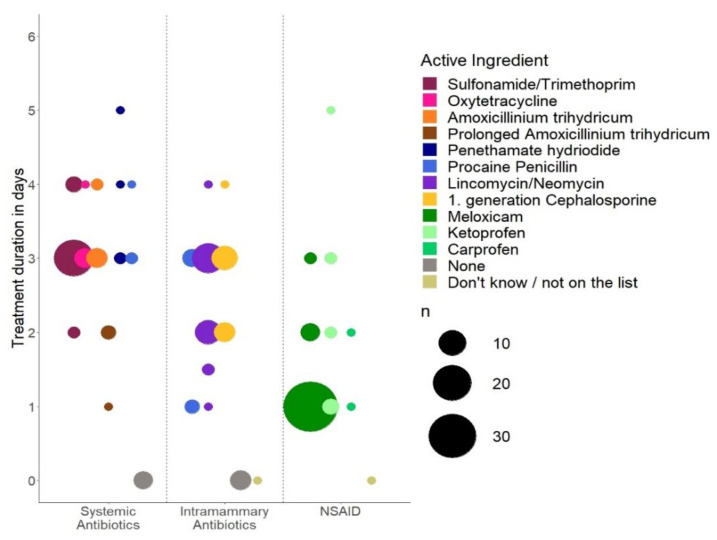
Prescription protocol of systemic antibiotics, local antibiotics and NSAIDs used often or always by Danish veterinarians (*n* = 58) for a herd diagnosis of “acute mastitis”.

Only veterinarians who were responsible for VASCs with a module 2 were eligible to answer these questions (*n* = 80). To get an estimate of a generalizable example, respondents were asked whether they generally prescribe the same treatment protocol for “common mastitis” and “acute mastitis” across herds. Respondents who replied that they used the same treatment protocol often or always (as opposed to “sometimes”, “rarely” or “never”) for the two types of diagnosis were then asked to describe their most used treatment protocol. Based on these conditions, *n* = 70 and *n* = 58 of the respondents were asked to describe their most used treatment protocol for “common mastitis” and “acute mastitis”, respectively. The treatment protocols are illustrated in a dotplot in Figure 2 and Figure 3 with abbreviated answers, and the full answers can be found in the Appendix A, Table A1, Table A2 and Table A3. The *x*-axis groups substances into systemically and locally (intramammary) administered antibiotics and nonsteroidal anti-inflammatory drugs (NSAIDs), and the *y*-axis shows the duration of treatment in days. The color of the dots indicates the specific active substance, while the size of the dot indicates the number of respondents who chose the given combination of substance and duration. Almost all prescription protocols included all three substances, i.e., treatments consisted of systemically as well as locally administered antibiotics in combination with NSAIDs. The most popular treatment protocol for “common mastitis” was combined systemic and local treatment with procaine penicillin for 3 days and supportive treatment with meloxicam for 1 day. The most popular treatment protocol for “acute mastitis” consisted of 3 days of systemic treatment with sulfonamide/trimethoprim combinations and local treatment with lincomycin/neomycin combinations, as well as supportive treatment with meloxicam for 1 day. A small number of respondents (*n* = 5 for systemic and *n* = 6 for local administration) stated that their first choice did not contain antibiotic treatment at all in cases of “acute mastitis”.

## 3. Discussion

### 3.1. Considerations in the Decision-Making Process

There was a strong emphasis on the severity of clinical signs when the respondents were asked when and why they chose to initiate antibiotic treatment. Even if this might seem obvious to some, it is noteworthy that the legislative framework in Denmark is based mainly on paraclinical information, such as milk sample analysis, as opposed to an evaluation that includes clinical factors—which the practitioners appear to use extensively. It makes sense to account for the severity of clinical symptoms when initiating treatment, and it is particularly important due to animal welfare considerations. However, given that a small percentage of respondents stated that they often or always treat even mild cases, it is also worth considering that not all cases of mastitis benefit from antibiotic treatment and that self-cure is relatively likely in some cases [4].

### 3.2. Route of Administration

The most widely used route of administration for antibiotic treatment in cases of clinical mastitis in Denmark was simultaneous systemic and local treatment. Studies from other countries show that preferences about the route of administration can vary. The Danish approach of combined treatment is very different to those highlighted by studies from other countries. In the United States, for instance, the standard approach seems to be the use of local treatment only [13,14]. Even other Scandinavian countries such as Sweden may have different approaches. According to a similar survey, Swedish veterinarians mostly use only systemic treatment in cases of clinical mastitis [15]. It is difficult to infer whether this is the best approach, since studies on the routes of administration are sparse and generally differ in terms of the choice of substance as well as the pathogenic involvement [16,17]. A study from 2014 showed no difference in the outcome following treatment with either locally or systemically administered penicillin over 5 days in cases of mastitis caused by Gram-positive penicillin-sensitive agents [18]. From a pharmacological perspective, it makes sense that there should be differences in the applicability of different substances due to their chemical properties and ability to reach the site of infection after either systemic or local application [19]. Regarding mastitis cases caused by Gram-negative infections, specifically *E. coli*, several studies show that local antibiotic treatment is actually not indicated, and only severe cases might benefit from systemic antibiotics. In these cases, limiting the total amount of unnecessary antibiotic use should be emphasized [20,21]. There is evidence from recent studies that systemic treatment may contribute to a higher risk of developing more antibiotic resistance [22]. That would be a reason to argue that treatment strategies should focus more on the potential of local administration only for mastitis cases caused by Gram-positive bacteria in the future.

### 3.3. Use of NSAIDs

The use of NSAIDs as a supportive measure in all cases of clinical mastitis is strongly recommended in the literature [23,24]. The current study indicates that this key point seems to be the biggest success in the practical application of evidence. Almost all the responding veterinarians said that they would often or always use “nonsteroidal anti-inflammatory drugs (NSAIDs) in combination with antibiotic treatment”. Likewise, the majority state that they would use NSAIDs alone. A survey of Swedish veterinarians’ treatment practice of clinical mastitis also reported that the majority of their treatments contain NSAIDs [15]. The use of NSAIDs is recommendable, considering that mastitis is known to be a painful and stressful disease and it is particularly important in cases where Gram-negative agents are involved, due to the limited added value of antibiotic treatment [20].

### 3.4. Milk Sampling, Treatment Initiation and Pathogen-Specific Targeting

Statutory requirements are known to be an important driver of diagnostic testing in animal production in Britain [25]. Likewise, the majority of the Danish cattle practitioners in the present study indicated that legislation was an important factor for obtaining milk samples. Similar to Swedish dairy practitioners [15], our results also indicate that Danish veterinarians were willing to collect milk samples for other reasons not related to legislation. This is a positive finding in relation to evidence-based mastitis treatment and implies that knowledge of the causative agent is of great importance for making the best treatment decisions [4,23]. However, as mentioned, most cases of clinical mastitis are not handled by a veterinarian, but by trained farm employees. The questions in this survey were directed specifically at the veterinarians, so the answers are not expected to reflect whether milk samples are taken by farm employees when handling cases themselves. Furthermore, when asked about the point at which they initiated treatment, a clear majority stated that they did so before they had the results from the milk samples. Even so, a few respondents stated that they would always initiate treatment after they received the results. This appears to be conflicting, yet there could be several ways to interpret this. The very nature of the current technology used to analyze milk samples in Denmark (traditional bacterial culture and commercially available PCR test) means that veterinarians will not receive the results until approximately 24–48 h later. Implementing newer technology, such as on-farm testing, could alleviate this problem [26]. It may not be feasible to use the results of a specific case with the current methods, unless there is a willingness to wait this long and risk that the cow’s clinical symptoms might worsen in the meantime. However, the next question about treatment targeting revealed that the majority use “knowledge of previous cases of mastitis at the same farm” to target their treatment. “Knowledge of the bacteriological state of the herd” also commonly influenced decisions about whether treatment should be initiated. This might mean that even though specific test results might become available too late to influence the decision about treatment initiation and choice of substance for one specific case, veterinarians will still use them as a surveillance tool for the herd, in order to inform future decisions on the treatment of clinical cases. Furthermore, practitioners are obligated to adjust the initial treatment when gaining new knowledge through the causative pathogen analysis, but the delay and possibility of unsuitable treatment in the meantime remain. Similar findings have been reported by other researchers. For example, “*history of response on that farm*” and “*on-farm disease pattern*” were the factors most frequently selected as influencing the prescriptions made by cattle practitioners in New Zealand [27], and “*experience of clinical efficacy in a given herd*” was the most influential factor for Danish swine practitioners in terms of drug choice for intestinal diseases [28]. The “*knowledge of previous infectious agents in the herd*” often affected Swedish veterinarians’ choice of antimicrobial therapy for bovine mastitis [15]. Overall, the present and previous studies indicate that herd health practitioners might be greatly influenced by previous microbiological findings and experience of treatments within the given herd when prescribing antimicrobials. The answers given also indicate that clinical signs and the appearance of the milk play a role for many of the respondents in building their suspected etiology. While this may be a widely used approach, it should be stressed that there is no scientific evidence to support it [4].

### 3.5. Veterinarian Protocols for Treatments Conducted by Farm Employees

The examples of treatment protocols, for the two types of herd diagnosis mostly used for clinical mastitis in Denmark, illustrate and underline the previously discussed general inclination toward combined administration. The Danish guidelines encouraging the use of simple penicillins appear to have been widely adopted by veterinarians when looking at prescriptions for common mastitis. In these protocols, procaine penicillin and penethamate hydriodide dominate within the systemic treatments. Likewise, procain penicillin dominates within the local treatments. According to a randomized field trial, systemic administration of penicillin in the form of penethamate hydroiodide is effective against some agents [29]. In general, the use of simple penicillins for mastitis caused by Gram-positive bacteria is supported by the literature; however, there are limitations due to variation in the susceptibility [23]. The current method for treating suspected Gram-negative cases, exemplified by the “acute mastitis” protocols, has a clear potential for improvement in relation to lowering the amount of antibiotic usage. This could be facilitated by refraining from antibiotic treatment in Gram-negative mastitis cases of mild–moderate severity, as suggested by the evidence presented in the literature [20,21,26]. However, the application depends on timely identification of the mastitis-causing bacteria. In addition to the two main categories of herd diagnosis previously mentioned, there are opportunities to use alternative prescription protocols in the Danish setting (e.g., only supportive treatment for expected Gram-negative cases with mild–moderate clinical symptoms). It is not known what stage of clinical severity the reported protocols reflect and it is possible that such a differential practice for mild cases already exists. However, since at least the very severe cases require a veterinarian’s attendance, as they are the only ones with the authority to give antibiotics intravenously, it is presumed that the reported protocols are not aimed for these.

### 3.6. Strengths and Limitations

The present study utilized register data for initial identification of the study population. This method should have a relatively low risk of bias compared to other common methods such as membership lists or conference participants. The demographic information from the respondents reflects that they are reasonably representative of the population of Danish cattle veterinarians [30]. The use of web-based surveys to assess populations is a common practice that is widely accepted as a useful tool in investigating general tendencies, despite its known limitations [31]. Piloting the questionnaire and involving additional experts in the field should have ensured that the survey was of a reasonable quality. Regardless, all questionnaire-based surveys carry a risk of information and self-reporting bias [32], which could mean that respondents would be inclined to answer how they think they should, instead of remaining completely objective, also known as social desirability bias. Although being able to answer anonymously should lower the impact of this bias, it should always be kept in mind when interpreting the results of surveys.

## 4. Material and Methods

### 4.1. Study Population

**Figure 4:**
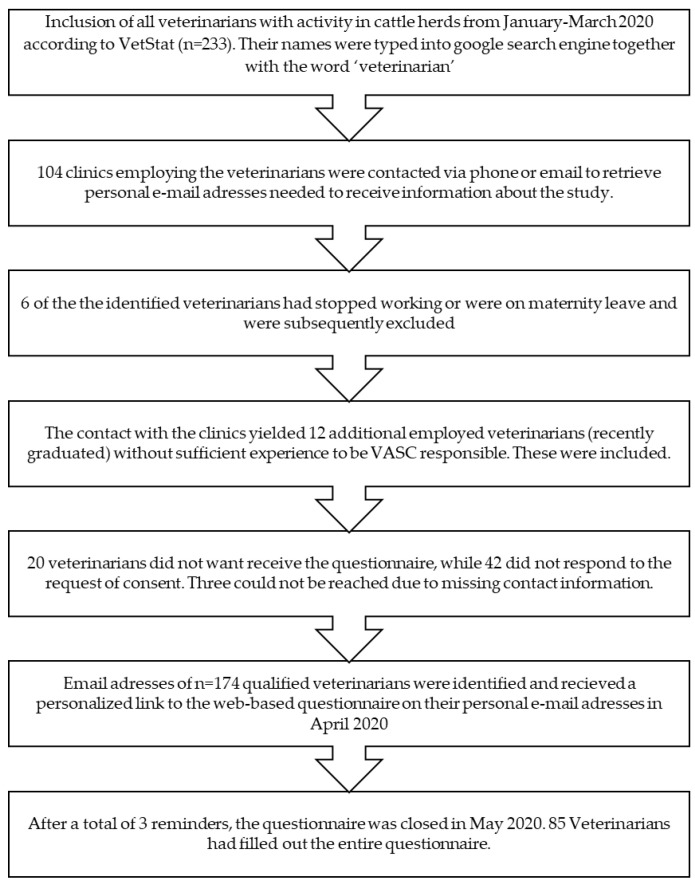
Flowchart of the selection process of the study population for the questionnaire survey of veterinarian treatment approach and antibiotic usage for mastitis by Danish cattle veterinarians.

The studied population was Danish veterinarians prescribing antibiotics for mastitis in Danish dairy herds. The study population was selected using register data from “VetStat” (the official Danish surveillance database of veterinary prescriptions and activity [12,33]) as previously suggested [28]. Permission to handle the personal data involved in the process was granted by the Danish Data Protection Agency. Following permission from the Danish Veterinary and Food Administration specifically for the purpose of this study, we extracted a list of all veterinarians who had at least one VASC relating to cattle in March 2020 from the “VetStat” database, based on the knowledge that this condition applies for the absolute majority of veterinarians working with cattle. Registered prescriptions relating to cattle udder health issued between January and March 2020 were used to validate the current activity of veterinarians with very few VASCs (≤2). As a next step, we contacted all clinics by e-mail or phone in order to retrieve the email addresses of the relevant veterinarians, in cases where they were not available directly online. We also asked them whether they had any recently graduated veterinarians working with cattle employed, since they are usually not responsible for VASCs yet and would thus have been missed by our first screening. The whole process is described in Figure 4.

### 4.2. Questionnaire Design

Questions were developed according to the guidelines provided by D.H. Stone [34] and aimed to cover a range of decisions that are indispensable during the course of clinical mastitis treatment. This study focuses on veterinary decision-making processes and routines at the time of diagnosis and initiation of treatment for clinical mastitis. The suggested optional answers were based on previous studies on the motivation behind treatment decisions in Denmark [35] and surveys conducted in other countries on similar topics [15,36,37,38,39]. All questions were discussed by a panel consisting of the authors, and two other researchers working on similar topics for their input. When all the questions were formulated, they were piloted by three cattle practitioners from different clinics. Their feedback was also included in the final formulation. The original questionnaire was conducted in Danish and can be obtained from the first author.

### 4.3. Types of Questions

In order to determine the demographic range, the introduction featured a series of simple questions about age, gender, geographic location, experience with cattle and treatment frequency for clinical mastitis. Following the introduction, there were two main types of questions, with answers given on a closed 5-point Likert scale with the options: “always”, “often”, “sometimes”, “rarely” and “never” (e.g., *How often do you take milk samples of clinical mastitis prior to initiating a treatment?*), or semi-open with a list of multiple-choice options and the option to add other answers. The semi-open questions were about the motivation behind decisions (why-questions) or practical circumstances (how-questions). For example, a why-question might pertain to the basis on which the choice of antibiotic substance was made, with a list of pharmacological (e.g., knowledge about the drug from the literature) and non-pharmacological (e.g., the farmer’s opinion) reasons, including an option of “don’t know” and a blank field for other suggestions. The respondents could choose how many options they wanted to list in their answer. An example of a how-question might be to describe the most used prescription protocol for a given diagnosis of “common” or “acute” clinical mastitis in a module 2 herd. To answer this, the respondents could choose from a list of pharmacological substances available in Denmark and write the duration in number of days.

### 4.4. Format and Data Collection

All responses were anonymized prior to analysis. The online platform used for this survey was SurveyXact Version 13.1 (Rambøll management consulting). To accommodate the respondents, certain questions were only shown based on previous answers, meaning that only relevant follow-up questions were available. All visible questions required completion. The retrieved e-mail addresses were uploaded, and distribution was managed via the platform. Participants who had not yet responded were sent reminders after 2 and 4 weeks. The total data collection took place over 5 weeks from April to May 2020. After completion, all collected data were extracted and stored in a secure hard drive, provided by the University of Copenhagen. Personal information was stored in the same way.

### 4.5. Data Analysis

Completed questionnaires were imported and analyzed in the statistical software “R” Version 4.0.0 [40]. Data structuring was facilitated by the “tidyverse” package [41] and illustrations for descriptive statistics were produced using the “ggplot2” package [42] and Microsoft Excel [43].

### 4.6. Descriptive Statistics

For questions that were answered by all 85 respondents, the percentage of the whole was calculated for each option. As mentioned, it was possible to choose multiple answers from a list for some of the questions. In these cases, it is important to note that the sum of respondents for all possible answers will not be equal to 100%, as respondents will most probably be represented in multiple options. The answer options are arranged according to popularity and do not reflect the original order.

## 5. Conclusions

The Danish veterinarians’ approach to the treatment of clinical mastitis and antibiotic usage is characterized by simultaneous systemic and local treatment. Their treatment decisions are generally based on clinical severity and a herd history of pathogenic involvement. The supportive use of NSAIDs is also widely used as a supplement to antibiotic treatment. The legislative framework works well in encouraging veterinarians to take milk samples for pathogen analysis, yet the current methods used do not work quickly enough for direct application of the knowledge gained. The implementation of more rapid diagnostic methods could help in solving this issue to some degree. Once this has been addressed, the treatment of Gram-negative cases could benefit from revision to follow the most recent knowledge on best practice. More studies on the potential to treat clinical mastitis solely via intramammary administration of antibiotics are needed in order to further improve strategies for the prudent use of antibiotics.

## Figures and Tables

**Table 1:** Number (*n*) and proportion (%) of respondents who marked the suggested answers often considered when making decisions about initiating treatment. Based on answers from 85 Danish veterinarians.

Question: What Aspects Do You Generally Consider When Making Decisions about Initiating Treatment for Clinical Mastitis? (Choose One or More Suggested Answers)		
List of Suggested Answers	*n*	Proportion of Total
Clinical signs in milk and udders	81	95%
General condition of the cow	81	95%
Knowledge of the bacteriological state of the herd	56	66%
Factors related to the cow (e.g., parity, time-point during lactation, mastitis history)	52	61%
Cell count history	32	38%
The herd manager’s opinion	26	31%
Current cell count	14	17%
Herd-related factors (e.g., rate of new infections, cure rate)	14	17%
Other (open answers)	5	6%

**Table 2:** Number (*n*) and proportion (%) of respondents who marked the suggested factors identified when making decisions about collecting milk samples in cases of clinical mastitis. Based on answers from 85 Danish veterinarians.

Question: On Which Factors Do You Base Your Decision about Whether or Not to Collect Milk Samples for Laboratory Diagnostics? (Choose One or More Suggested Answers)		
List of Suggested Answers	*n*	Proportion of Total
Legislation	62	73%
Severity of clinical signs	44	52%
The herd manager’s opinion on sample analysis	35	41%
Other (open answers)	33	39%
Visual appearance, consistency or smell of the milk	30	35%
Knowledge of the bacteriological state of the herd	23	27%
Factors related to the cow (e.g., parity, time-point during lactation, mastitis history)	16	19%
Herd type (e.g., conventional/organic)	14	17%
Cell count registrations	6	7%
None of the above	6	7%
Don’t know	0	0%

**Table 3:** Number (*n*) and proportion (%) of respondents who marked the suggested answers about indications used for targeting treatment toward specific pathogens in cases of clinical mastitis. Based on answers from 85 Danish veterinarians.

Question: When You Target the Initial Treatment Strategy toward a Specific Pathogen before Laboratory Results Are Available, What Factors Do You Base This on? (Choose One or More Suggested Answers)		
List of Suggested Answers	*n*	Proportion of Total
Severity of clinical signs	79	93%
Visual appearance, consistency or smell of the milk	73	86%
Knowledge about the pathogen involved in previous cases of mastitis within the same herd	58	68%
The rate at which clinical signs appear	49	58%
Experience of the effect of the treatment within the same herd	49	58%
Knowledge about the pathogen involved in previous cases of mastitis in the same cow	19	22%
The herd manager’s opinion	7	8%
Other (open answers)	4	5%
Don’t know	0	0%
Never target treatment toward a specific pathogen	2	2%

## Data Availability

The data presented in this study are available on request from the corresponding author. The data are not publicly available due to privacy.

## Appendix A

**Table A1:** A full list of all possible choices in questions about treatment protocol, based on available products on the Danish market.

**Systemic**	Procaine Penicillin (e.g., Penovet)
	Penethamate hydriodide (e.g., Mamyzin)
	Amoxicillinium trihydricum (e.g., Curamox)
	Prolonged Amoxicillinium trihydricum (e.g., Curamox prolungatum)
	Benzylpenicillin/Dihydrostreptomycin (e.g., Streptocillin)
	Sulfonamide Trimethoprim combinations (e.g., Norodine)
	Oxytetracycline (e.g., Alamycin)
	Tylosin (e.g., Tylan)
	3rd-4th generation Cephalosporine (e.g., Cobactan)
	Fluoroquinolon (e.g., Baytril)
	First choice of treatment does NOT contain systemic antibiotics
	Don’t know/not on the list
**Local**	Procaine Penicillin (e.g., Carepen)
	Lincomycin/Neomycin (e.g., Albiotic)
	Benzylpenicillin/Dihydrostreptomycin (e.g., Streptocillin intramammarium)
	1st generation Cephalosporine (e.g., Rilexine)
	3rd-4th generation Cephalosporine (e.g., Cobactan intramammarium)
	First choice of treatment does NOT contain local antibiotics
	Don’t know/not on the list
**NSAID**	Ketoprofen (e.g., Romefen)
	Carprofen (e.g., Rimadyl)
	Meloxicam (e.g., Metacam)
	First choice of treatment does NOT contain NSAIDs
	Don’t know/not on the list

**Table A2:** Distribution of respondents treatment choice and duration in days for systemic antibiotics, local antibiotics and NSAIDs used often or always by Danish veterinarians (*n* = 58) for a herd diagnosis of “acute mastitis”.

Original Question, Translated		Number of Respondents Per Option						
Describe the treatment protocol that you use (often or always) and choose the corresponding number of days for the herd diagnosis of ‘acute mastitis’ here:								
		Days /0	1	1.5	2	3	4	5
Systemic antibiotics	Procaine Penicillin (e.g., Penovet)					2	1	
	Penethamate hydriodide (e.g., Mamyzin)					2	1	1
	Amoxicillinium trihydricum (e.g., Curamox					6	2	
	Prolonged Amoxicillinium trihydricum (e.g., Curamox prolungatum)		1		3			
	Sulfonamide Trimethoprim combinations (e.g., Norodine)				2	21	4	
	Oxytetracycline (e.g., Alamycin)					6	1	
	First choice of treatment does NOT contain systemic antibiotics	5						
Local antibiotics	Procaine Penicillin (e.g., carepen)		3			5		
	Lincomycin/Neomycin (e.g., Albiotic)		1	2	9	14	1	
	1. generation Cephalosporine (e.g., Rilexine)				6	9	1	
	First choice of treatment does NOT contain local antibiotics	6						
	Don’t know/not on the list	1						
NSAID	Ketoprofen (e.g., Romefen)		4		2	2		1
	Carprofen (e.g., Rimadyl)		1			1		
	Meloxicam (e.g., Metacam)		39		5	2		
	Don’t know/not on the list	1						

**Table A3:** Distribution of respondents treatment choice and duration in days for systemic antibiotics, local antibiotics and nonsteroidal anti-inflammatory drugs (NSAIDs) used often or always by Danish veterinarians (n = 70) for a herd diagnosis of “common mastitis”.

Original Question, Translated		Number of Respondents Per Option					
Describe the treatment protocol that you use (often or always) and choose the corresponding number of days for the herd diagnosis of ‘common mastitis’ here:							
		Days /0	1	2	3	4	5
Systemic antibiotics	Procaine Penicillin (e.g., Penovet)				26	12	11
	Penethamate hydriodide (e.g., Mamyzin)				11	7	2
	Amoxicillinium trihydricum (e.g., Curamox)					1	
Local antibiotics	Procaine Penicillin (e.g., carepen)		17	15	35	9	1
	Lincomycin/Neomycin (e.g., Albiotic)					1	
	First choice of treatment does NOT contain local antibiotics	1					
	Don’t know/not on the list	1					
NSAID	Ketoprofen (e.g., Romefen)		2		2		
	Carprofen (e.g., Rimadyl)		1				
	Meloxicam (e.g., Metacam)		59	1	1		
	First choice of treatment does NOT contain NSAID	2					
	Don’t know/not on the list	2					

**Table A4:** Answers from 85 Danish veterinarians to closed questions about the use of supportive treatment, other than NSAIDs, in cases of clinical mastitis.

How Often Do You (or the Farmer) Use the Following Other Supportive Treatment Options in Cases of Clinical Mastitis?					
	Always	Often	Sometimes	Rarely	Never
Glucocorticoids	0.0%	2.4%	14.1%	27.1%	56.5%
Oxytocin	0.0%	5.9%	35.3%	40.0%	18.8%
Calcium SC/IV	0.0%	14.1%	72.9%	10.6%	2.4%
Hypertonic solutions IV (saltwater)	0.0%	9.4%	51.8%	21.2%	17.6%
Oral electrolytes	0.0%	11.8%	50.6%	18.8%	18.8%
Oral fluids	0.0%	28.2%	56.5%	11.8%	3.5%
Others	0.0%	4.7%	25.9%	31.8%	37.6%
